# O-GlcNAc elevation through activation of the hexosamine biosynthetic pathway enhances cancer cell chemoresistance

**DOI:** 10.1038/s41419-018-0522-0

**Published:** 2018-04-30

**Authors:** Yubo Liu, Yu Cao, Xiaoqing Pan, Meiyun Shi, Qiong Wu, Tianmiao Huang, Hui Jiang, Wenli Li, Jianing Zhang

**Affiliations:** 1 School of Life Science and Medicine, Dalian University of Technology, Panjin, China; 2 School of Life Science and Biotechnology, Dalian University of Technology, Dalian, China

## Abstract

Chemoresistance has become a major obstacle to the success of cancer therapy, but the mechanisms underlying chemoresistance are not yet fully understood. O-GlcNAcylation is a post-translational modification that is regulated by the hexosamine biosynthetic pathway (HBP) and has an important role in a wide range of cellular functions. Here we assessed the role of O-GlcNAcylation in chemoresistance and investigated the underlying cellular mechanisms. The results showed that the HBP has an important role in cancer cell chemoresistance by regulating O-GlcNAcylation. An increase in the levels of O-GlcNAcylation indicates an increased resistance of cancer cells to chemotherapy. Acute treatment with doxorubicin (DOX) or camptothecin (CPT) induced O-GlcNAcylation through HBP activation. In fact, the chemotherapy agents activated the AKT/X-box-binding protein 1 (XBP1) axis and then induced the HBP. Furthermore, the observed elevation of cellular O-GlcNAcylation led to activation of survival signalling pathways and chemoresistance in cancer cells. Finally, suppression of O-GlcNAcylation reduced the resistance of both established and primary cancer cells to chemotherapy. These results provide significant novel insights regarding the important role of the HBP and O-GlcNAcylation in regulating cancer chemoresistance. Thus, O-GlcNAc inhibition might offer a new strategy for improving the efficacy of chemotherapy.

## Introduction

Chemotherapy is one of the standard treatment methods for many cancers and the development of chemoresistance, either intrinsic or acquired, is the most commonly encountered phenomenon that limits the success of cancer chemotherapy^1,2^. Understanding the mechanisms through which chemoresistance occurs has huge implications for potentiating the cancer cell-killing effect of chemotherapy. The chemoresistance of cancer cells involves complicated mechanisms, including the overexpression of multidrug efflux transporters, such as P-glycoprotein (P-gp), the activation of pro-survival pathways and ineffective induction of cell death^2^. However, the mechanisms modulating chemoresistance in cancers are not completely clear.

A growing body of evidence demonstrates that cancer metabolic reprogramming might influence the expression of genes to drive oncogenesis and maintain cell viability in response to stress, including drug treatment^3,4^. For example, the glycolytic metabolism not only alters transcription but also affects the repair of DNA damage by having an impact on the global chromatin structure in cancer cells^5,6^. Most malignant tissues have increased glucose uptake associated with increased rates of glycolysis and glucose transport^7^. Even though the majority of glucose enters glycolysis, ~ 2–5% of glucose influx is directed toward the hexosamine biosynthetic pathway (HBP). This pathway generates UDP-GlcNAc, which is a nucleotide sugar substrate involved in multiple biological processes, including classical glycosylation and O-GlcNAcylation^8,9^. The available evidence suggests that alteration of the pool of activated substrates might lead to aberrant O-GlcNAcylation^10^. Thus, the HBP might link the altered cancer metabolism with aberrant glycosylation, providing a mechanism for how cancer cells sense and respond to a variety of cellular stress, including chemotherapy.

O-GlcNAcylation is a dynamic and reversible glycosylation of serine or threonine residues in a variety of nuclear and cytoplasmic proteins. The addition of O-GlcNAc to proteins is catalysed by O-GlcNAc transferase (OGT) and its removal is catalysed by O-GlcNAcase (OGA). As a post-translational modification, O-GlcNAcylation regulates a wide range of cellular functions. In response to numerous forms of cellular stress or injury, including DNA damage, the O-GlcNAcylation levels are dynamically elevated in both in vitro and in vivo models^9,11,12^. Many O-GlcNAcylated proteins bind double-stranded DNA-dependent protein kinase or double-stranded DNA breaks, suggesting a role for O-GlcNAcylation in regulating signalling pathways related to DNA damage repair and cell survival^11,13–15^. Together, these data indicate that HBP-induced O-GlcNAcylation might directly influence cell survival and resistance to DNA-targeting chemotherapy. However, the molecular mechanism(s) through which the HBP and O-GlcNAcylation regulate thresholds in cell death and enhance cell resistance have not been identified.

In this study, we investigated the relevant role of the HBP and O-GlcNAcylation in the route leading to cancer cell resistance to chemotherapy and obtained novel mechanistic data demonstrating that chemotherapy induces flux through the HBP and then elevates cellular O-GlcNAcylation, resulting in the activation of survival-related signalling pathways and chemoresistance in cancer cells. The findings demonstrate that the combination of chemotherapy with O-GlcNAcylation inhibition bypasses chemoresistance in both established and primary cancer cells.

## Results

### Level of protein O-GlcNAcylation correlates with the cellular response to chemotherapy

**Fig. 1:**
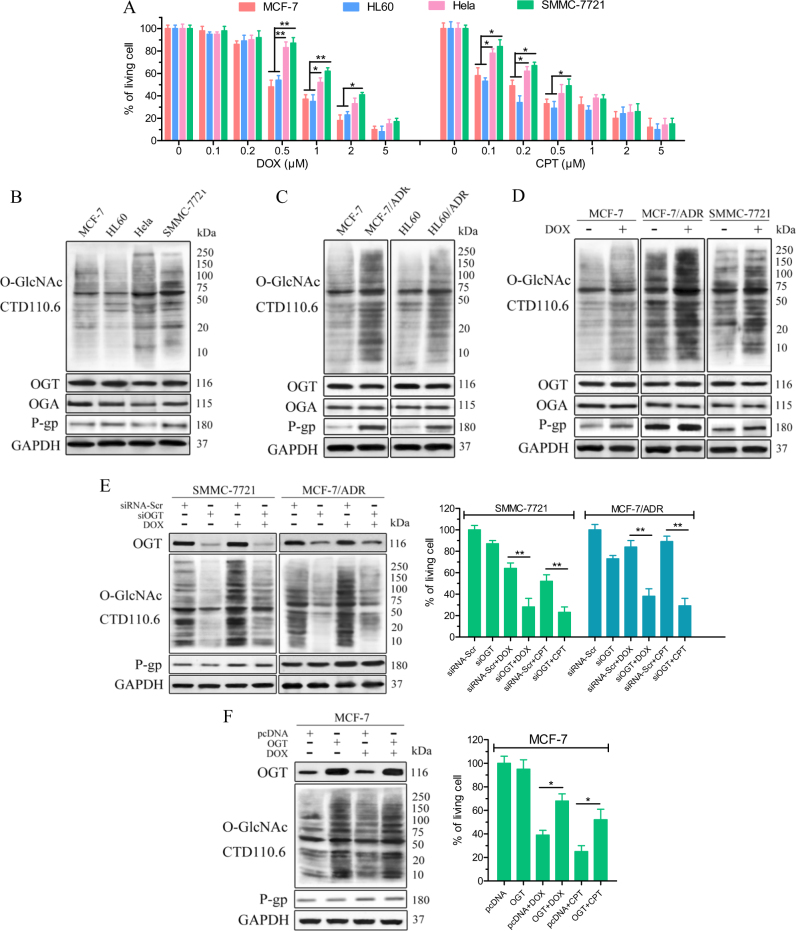
A high level of protein O-GlcNAcylation is associated with chemoresistance in cancer cell lines. **a** MCF-7, HL60, Hela and SMMC-7721 cells were treated with the indicated dose of DOX or CPT for 24 h. The cell viability was assessed through the MTS assay. **b** and **c** Cells with intrinsic and acquired chemoresistance contain elevated levels of O-GlcNAc-modified proteins compared with sensitive cells. The cellular O-GlcNAcylation and expression of indicated proteins in parental and resistant cancer cell lines were analysed by immunoblotting analysis. **d** O-GlcNAcylation is upregulated in both parental and resistant cancer cells after transient treatment with DOX. The indicated cells were treated with DOX (0.1 μM for MCF-7, 1 μM for MCF-7/ADR and SMMC-7721) for 6 h. Whole-cell lysates were obtained after treatment and analysed by immunoblotting. **e** The indicated cell lines were transfected with scrambled siRNA (siRNA-Scr) or OGT siRNA (siOGT) for 48 h and then treated with 1 μM DOX or 0.5 μM CPT for an additional time. The protein levels after treatment with DOX or CPT for 6 h were examined by immunoblotting. The cell viability after treatment with DOX or CPT for 24 h was assessed through an MTS assay. **f** MCF-7 cells were transfected with control (pcDNA) or OGT-overexpressing vector (OGT) for 48 h and then treated with 1 μM DOX or 0.5 μM CPT for an additional time. The protein levels after treatment with DOX or CPT for 6 h were examined by immunoblotting. The cell viability after treatment with DOX or CPT for 24 h was assessed through an MTS assay. The data represent the means ± SEM, *N* = 3, *p < 0.05, **p < 0.01

We first investigated whether the protein O-GlcNAcylation levels contribute to the response of cells to chemotherapy. A panel of tumour cell lines (MCF-7, HL60, Hela and SMMC-7721) were treated with 0.1–5 μM doxorubicin (DOX) or camptothecin (CPT) for 24 h. As shown in Fig. 1a, Hela and SMMC-7721 cells exhibited more resistance than MCF-7 and HL60 cells. In agreement with our conjecture, the immunoblotting results showed that Hela and SMMC-7721 cells contained higher levels of O-GlcNAc-modified proteins than sensitive MCF-7 and HL60 cells (Fig. 1b). However, analysis of the enzymes that govern the O-GlcNAc moiety, OGT and OGA, revealed no clear correlation between the expression levels of these proteins and the cellular response to DOX and CPT. In addition, the multidrug resistance-related P-gp levels were also not directly associated with the cellular response to chemotherapy.

We also investigated whether O-GlcNAc modification participates in the acquired resistance of chemotherapy. DOX-resistant (ADR) variants were derived from MCF-7 and HL60 cells following continuous exposure to DOX for several months (Fig. S1A). Increases in cellular O-GlcNAcylation were observed in MCF-7/ADR and HL60/ADR cells compared with their parental cell lines (Fig. 1c). Although P-gp was upregulated, the expression levels of OGT and OGA remained constant across the parental and ADR sublines.

The O-GlcNAcylation levels showed moderate increases in MCF-7 cells and significant increases in MCF-7/ADR and SMMC-7721 cells upon acute treatment with DOX (Fig. 1d). Consistently, invisible changes in the OGT and OGA protein levels were observed in cells subjected to acute DOX. Similar results were obtained in SMMC-7721 and MCF-7/ADR treated with another frontline chemotherapeutic drug 5-Fluoracil (5-FU, Fig. S1B and S4A), indicating that the direct donor (UDP-GlcNAc) cellular levels of this modification was upregulated during drug stress. Further, our results showed that DOX-induced dynamic O-GlcNAcylation in resistant cells was decreased by OGT silencing (Fig. 1e). Correspondingly, OGT small interferig RNA (siRNA)-transfected cells showed increased sensitivity to DOX and CPT. In contrast, OGT overexpression in the parental MCF-7 cells reduced the levels of cell death in response to DOX (Fig. 1f). However, regardless of the OGT expression levels, P-gp can be markedly induced by DOX treatment in these cells, suggesting that O-GlcNAcylation potentiates chemotherapy resistance in a P-gp-independent manner.

In summary, these results indicate that an impact on the O-GlcNAcylation level protects cancer cells from cancer drug-induced cell death.

### The HBP is activated in cancer cells following exposure to chemotherapy agents

**Fig. 2:**
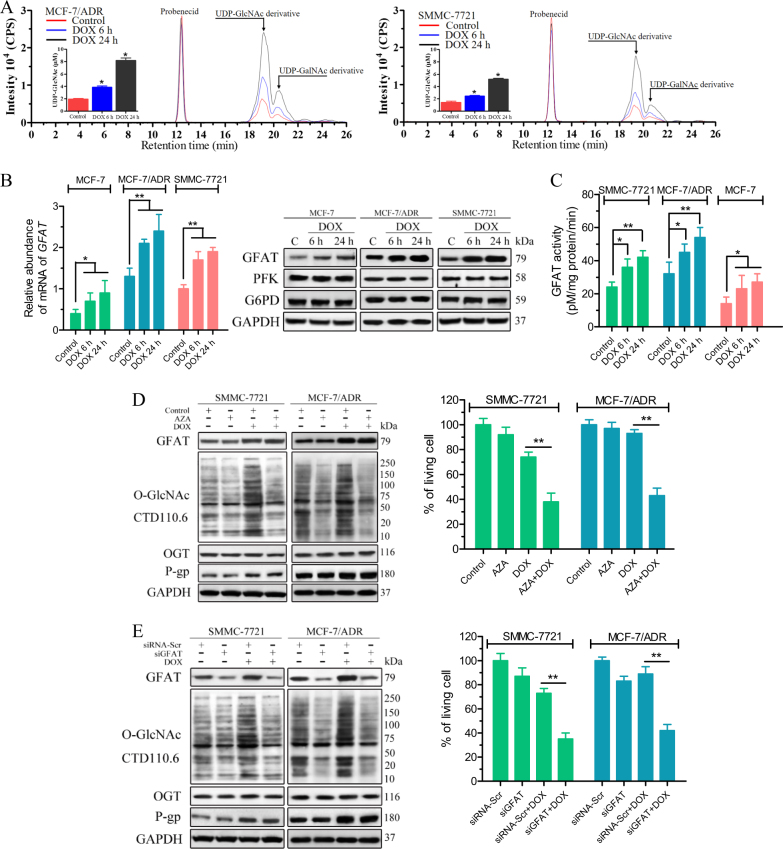
Flux through the HBP in cancer cells is up-regulated in response to drug stress. **a** DOX induces increases in the UDP-GlcNAc levels. UDP-GlcNAc in the cell lysates were derivatizated with trimethylsilyldiazomethane. Chromatograms of polar metabolites in MCF-7/ADR and SMMC-7721 cell extracts from control (red line), and 1 μM DOX-treated cells for 6 h (blue line) and 24 h (black line) show regions corresponding to the UDP-GlcNAc derivative, UDP-GalNAc derivative and probenecid retention times. Quantitative analyses are shown as means ± SEM, deviation of three independent experiments. *P*-values were calculated using one-way ANOVA and the appropriate post test. *P < 0.05. **b** The GFAT transcriptional and protein levels are upregulated in parental and resistant cell lines after transient treatment with DOX. MCF-7, MCF-7/ADR and SMMC-7721 cells were treated with DOX (0.1 μM for MCF-7, 1 μM for MCF-7/ADR and SMMC-7721) for indicated time and the GFAT transcript level was analysed by quantitative PCR. DMSO was used as a control. Whole-cell lysates were analysed by immunoblotting. **c** MCF-7, MCF-7/ADR and SMMC-7721 cells were treated with DOX (0.1 μM for MCF-7, 1 μM for MCF-7/ADR and SMMC-7721) for the indicated time and cell lysates were used for the analysis of GFAT activity. **d** SMMC-7721 and MCF-7/ADR cells were treated with 1 μM DOX alone or together with 5 μM AZA for 6 and 24 h. The protein levels after treatment with DOX or AZA for 6 h were examined by immunoblotting. DMSO was used as a control. The cell viability after treatment with DOX or AZA for 24 h was assessed through an MTS assay. **e** The indicated cell lines were transfected with scrambled siRNA (siRNA-Scr) or GFAT siRNA (siGFAT) for 48 h and then treated with 1 μM DOX. The protein levels after treatment with DOX for 6 h were examined by immunoblotting. The cell viability after treatment with DOX for 24 h was assessed through an MTS assay. The data represent the means ± SEM, *N* = 3, *p < 0.05, **p < 0.01

We then measured the cellular levels of UDP-GlcNAc and activation of the HBP after drug treatment. Through liquid chromatography, we were able to distinguish UDP-GlcNAc from UDP-GalNAc, and these distinct chemical species were further characterized by mass spectrum (MS) (Fig. 2a and S2). A progressive increase in the UDP-GlcNAc levels was observed in the DOX-treated MCF-7/ADR and SMMC-7721 cell extracts, and up to twofold (6 h) and fourfold (24 h) higher levels of UDP-GlcNAc were observed after DOX treatment compared with the untreated MCF-7/ADR cells. Similar results were observed in SMMC-7721 cells. We then evaluated the protein levels and activities of fructose-6-phosphate amidotransferase (GFAT), the key rate-limiting enzymes of the HBP^16^. The GFAT levels (messenger RNA and protein) were higher in SMMC-7721 and MCF-7/ADR cells than in MCF-7 cells (Fig. 2b). Further GFAT accumulation and increased GFAT activity were observed during DOX treatment in these cells (Fig. 2b,c). We also investigated the protein levels and activities of phosphofructokinase (PFK) and glucose 6-phosphate dehydrogenase (G6PD), which control the glucose metabolism through the glycolytic and pentose phosphate pathways, respectively^17^. No significant changes were observed after DOX treatment. Confirming a role for the HBP in drug resistance, GFAT inhibition with azaserine (AZA)^18^ significantly induced SMMC-7721 and MCF-7/ADR cell death under DOX treatment without affecting the expression of OGT and P-gp (Fig. 2d). In addition, the levels of O-GlcNAcylated proteins were reduced by GFAT inhibition and correspondingly similar results were obtained with GFAT siRNA (Fig. 2e). These results indicate upregulation of flux through the HBP during drug stress, revealing an uncharacterized regulation of the HBP in chemotherapy resistance.

### Activation of the AKT/XBP1 axis by chemotherapy regulates flux through the HBP

**Fig. 3:**
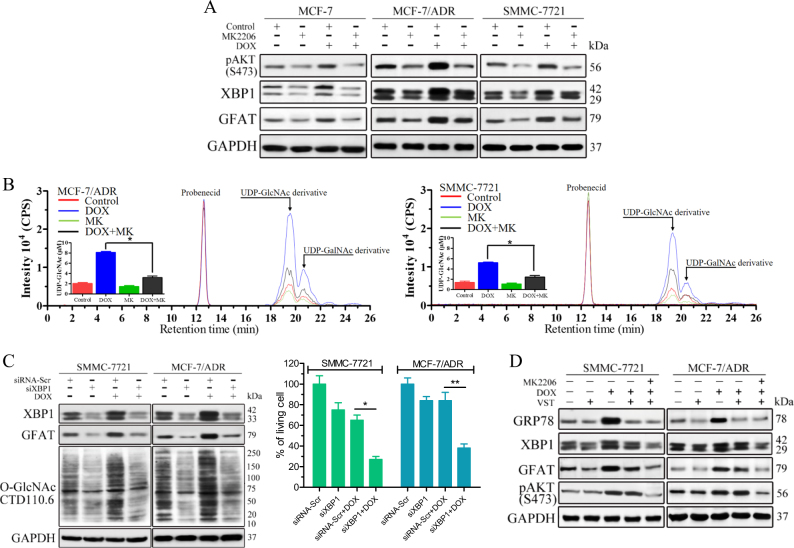
Chemotherapy-induced HBP activation is regulated by the AKT/XBP-1 axis. **a** AKT has a role in activating XBP1 and GFAT. The indicated cells were treated with DOX (0.1 μM for MCF-7, 1 μM for MCF-7/ADR and SMMC-7721) alone or together with 1 μM MK2206 for 6 h. DMSO was used as a control. The protein levels were examined by immunoblotting. **b**Induction of UDP-GlcNAc by DOX is attenuated by AKT inhibition. Chromatograms of polar metabolites in MCF-7/ADR and SMMC-7721 cell extracts from control cells (red line), cells treated with 1 μM DOX (blue line) or 1 μM MK2206 (green line) alone and cells treated with DOX plus MK2206 (black line) for 24 h show the regions corresponding to the UDP-GlcNAc derivative, UDP-GalNAc derivative and probenecid retention times. **c** XBP1 acts upstream of GFAT. The indicated cell lines were transfected with scrambled siRNA (siRNA-Scr) or XBP1 siRNA (siXBP1) for 48 h and then treated with 1 μM DOX. The protein levels after treatment with DOX for 6 h were examined by immunoblotting. The cell viability after treatment with DOX for 24 h was assessed through an MTS assay. **d** AKT/XBP1 axis regulates GFAT expression in a UPR-independent manner. The indicated cells were treated with 1 μM DOX alone or together with 3 μM VST for 6 h. DMSO was used as a control. The protein levels were examined by immunoblotting. The data represent the means ± SEM, *N* = 3, *p < 0.05, **p < 0.01

AKT has been implicated in regulation of the expression and activity of XBP1, which is the transcription factor of GFAT^19,20^. This study found that the phosphorylation of AKT on serine 473, a phosphatidylinositol-3-kinase (PI3K)-dependent target, was significantly higher in DOX-treated MCF-7/ADR cells than in MCF-7 cells, concomitant with the more efficient induction of X-box-binding protein 1 (XBP1) and GFAT (Fig. 3a). XBP1 splicing was also checked by reverse-transcriptase PCR (RT-PCR) (Fig.S3). Similarly, in SMMC-7721 cells, the AKT/XBP1 axis was also activated upon DOX treatment. AKT inhibitor MK2206^21^ abrogated the induction of XBP1 and GFAT by DOX treatment. In fact, a slight XBP1 induction was observed, indicating that other signalling pathways have roles in drug-induced HBP activation. Consistent results were observed in the cells treated with 5-FU (Fig. S4A). Furthermore, the induction of UDP-GlcNAc by DOX treatment in SMMC-7721 and MCF-7/ADR cells was also significantly attenuated by MK2206 (Fig. 3b), confirming that the inhibition of AKT compromises the potency of HBP activation.

SMMC-7721 and MCF-7/ADR cells were further treated with siRNA targeting XBP1. As expected, XBP1 acted upstream of GFAT and, as such, the downregulation of XBP1 reduced GFAT expression and O-GlcNAc modification of proteins in DOX-resistant cells (Fig. 3c). We also noticed an additional 30–40% cell death, which increased with the duration of the XBP1 siRNA treatment, compared with DOX treatment alone. To explore the role of unfolded protein response (UPR)^20^ in drug-induced HBP activation, the classic UPR inhibitor versipelostatin (VST) was used^22^ and the results showed that VST suppressed the accumulation of endogenous GRP78 (a commonly used indicator of UPR activation) in SMMC-7721 and MCF-7/ADR cells under DOX treatment. However, the downregulation of GRP78 expression partially inhibited XBP1 and downstream GFAT, indicating a UPR-independent mechanism for the regulation of XBP1 (Fig. 3d). Similar results were obtained with the knockdown of GRP78 using siRNA (Fig. S4B). Further analysis showed that pAKT (S473) was still inducible upon DOX treatment regardless of whether GRP78 was inhibited by VST or GRP78 siRNA. The combination of VST and MK2206 almost completely inhibited the induction of XBP1 and GFAT. These data provide compelling evidence that AKT activation by chemotherapy increases the flux through the HBP in a UPR-independent manner.

Taken together, these results suggest that activation of the HBP by chemotherapy could be a common mechanism through which cancer cells antagonize cell death and this mechanism is at least partially mediated through the AKT/XBP1 axis.

### Elevated O-GlcNAcylation of prosurvival proteins suppress cell death in response to chemotherapy

**Fig. 4:**
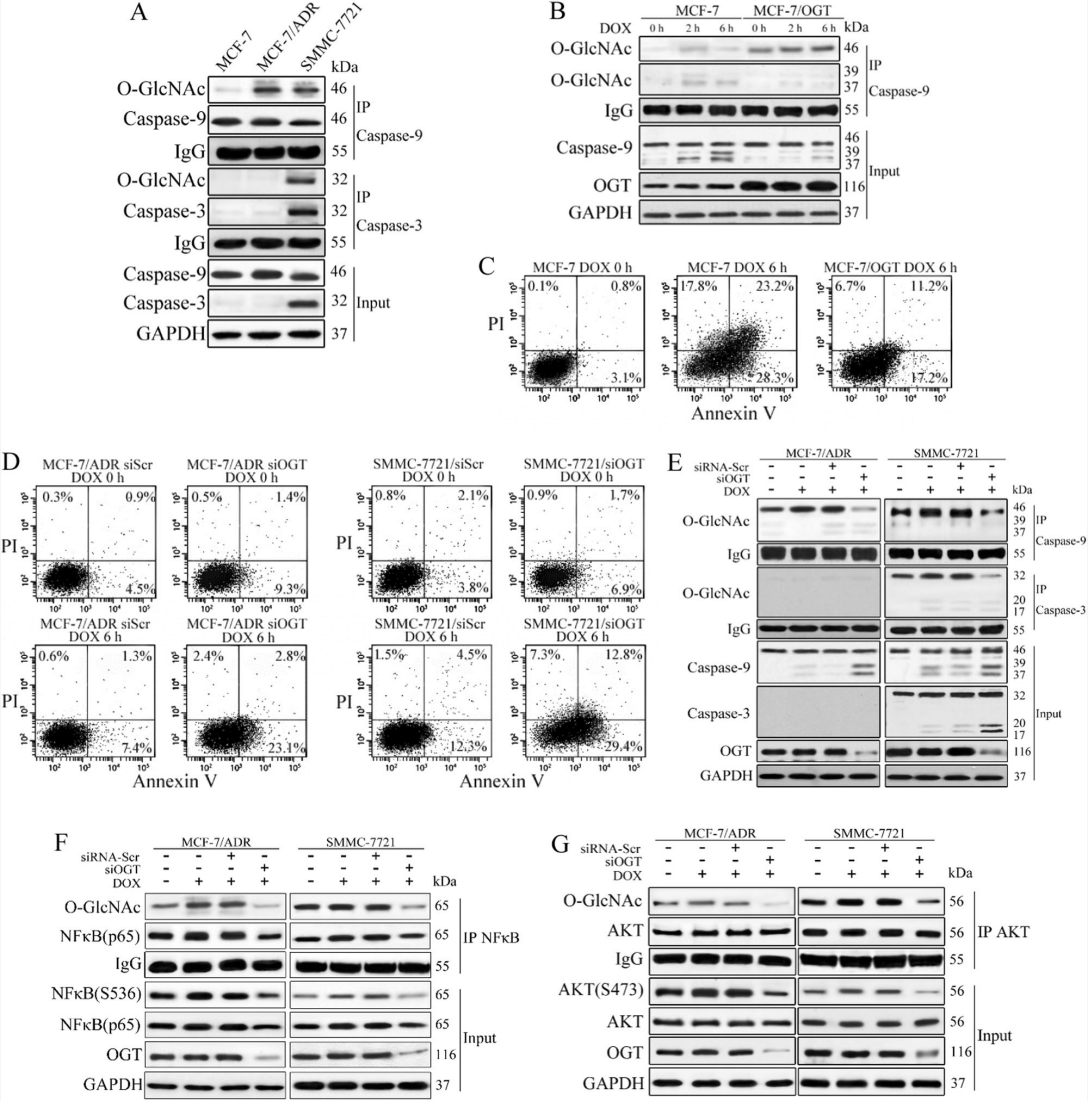
Chemotherapy-induced O-GlcNAcylation activates pro-survival pathways in cancer cells. **a** O-GlcNAcylation of caspase-9/3 is increased in resistant cells compared with sensitive MCF-7 cells. Caspase-9/3 immunoprecipitations were performed and the immunoprecipitated fractions were analysed by immunoblotting for O-GlcNAcylation. **b** Caspase-9 cleavage can be blocked by O-GlcNAcylation. MCF-7 cells were transfected with the control (pcDNA) or OGT-overexpressing vector (OGT) for 48 h and then treated with 1 μM DOX for the indicated time. Caspase-9 immunoprecipitation was performed and the immunoprecipitated fractions were analysed by immunoblotting for O-GlcNAcylation. **c** and **d** O-GlcNAcylation inhibits the apoptotic function of caspases. MCF-7 cells were stable transfected with the control (pcDNA3.1) or OGT-overexpressing vector (OGT) for 48 h and then treated with 1 μM DOX for the indicated time. MCF-7/ADR and SMMC-7721 cells were transfected with scrambled siRNA (siScr) or OGT siRNA (siOGT) for 48 h and then treated with 1 μM DOX for the indicated time, and apoptosis was then analysed through flow-cytometry with Annexin V/PI double staining. **e** MCF-7/ADR and SMMC-7721 cells were transfected with scrambled siRNA (siScr) or OGT siRNA (siOGT) for 48 h and then treated with 1 μM DOX for the indicated time. Caspase-3/9 immunoprecipitation was performed and the immunoprecipitated fractions were analysed by immunoblotting for O-GlcNAcylation. **f**and **g** The pro-survival transcription factors NF-κB and AKT were O-GlcNAcylated during DOX treatment. MCF-7/ADR and SMMC-7721 cells were transfected with scrambled siRNA (siRNA-Scr) or OGT siRNA (siOGT) for 48 h and then treated with 1 μM DOX for the indicated time. NF-κB and AKT immunoprecipitations were performed, and the immunoprecipitated fractions were analysed by immunoblotting

We next investigated the downstream targets of O-GlcNAcylation. A previous study found that the cleavage of apoptotic caspases could be blocked by O-GlcNAcylation^23^. In our study, the major intrinsic apoptotic caspases, namely caspase-3 and 9, were also O-GlcNAcylated in the parental and resistant cells. In MCF-7 and its resistant subline, O-GlcNAcylated caspase-3 was absent, because these are caspase-3-null cell lines. Notably, the O-GlcNAcylation of these caspases was increased in resistant SMMC-7721 and MCF-7/ADR cells compared with sensitive MCF-7 cells (Fig. 4a). Treatment of MCF-7 cells with DOX resulted in continuous caspase-9 activation with rare O-GlcNAcylation accumulation, whereas OGT overexpression decreased the activation rate and upregulated O-GlcNAcylated caspase-9 (Fig. 4b). Consistently, through Annexin V–fluorescein isothiocyanate (FITC)/propidium iodide (PI) staining, we found that the apoptosis rates of OGT-overexpressing cells were significantly decreased compared with the control cells in the presence of DOX (Fig. 4c). In resistant cells, DOX treatment led to further accumulation of O-GlcNAcylated caspase-9 (and caspase-3 in SMMC-7721 cells) and this effect was accompanied by slight caspase cleavage. In contrast, OGT silencing in the presence of DOX enhanced cell apoptosis and activation of caspase-9 and 3, indicating that the apoptotic function of caspases can be inhibited by chemotherapy-induced O-GlcNAcylation (Fig. 4d,e).

In addition, the pro-survival transcription factors nuclear factor-κB (NF-κB) and AKT were also O-GlcNAcylated during DOX treatment in resistant cells. OGT downregulation in SMMC-7721 and MCF-7/ADR cells decreased the NF-κB protein level and phosphorylation at Ser536, which is critical for NF-κB nuclear translocation and activation (Fig. 4f). Moreover, O-GlcNAcylation of AKT enhanced AKT phosphorylation at Ser473, indicating that AKT and the HBP are activated by a positive feedback mechanism involving O-GlcNAcylation (Fig. 4g). Based on the above results, we can conclude that chemotherapy-induced O-GlcNAcylation activates pro-survival pathways and strengthens the cell-death threshold of chemotherapy drugs.

### Combination of O-GlcNAc suppression with chemotherapy alleviates drug resistance in both established and primary cancer cells

**Fig. 5:**
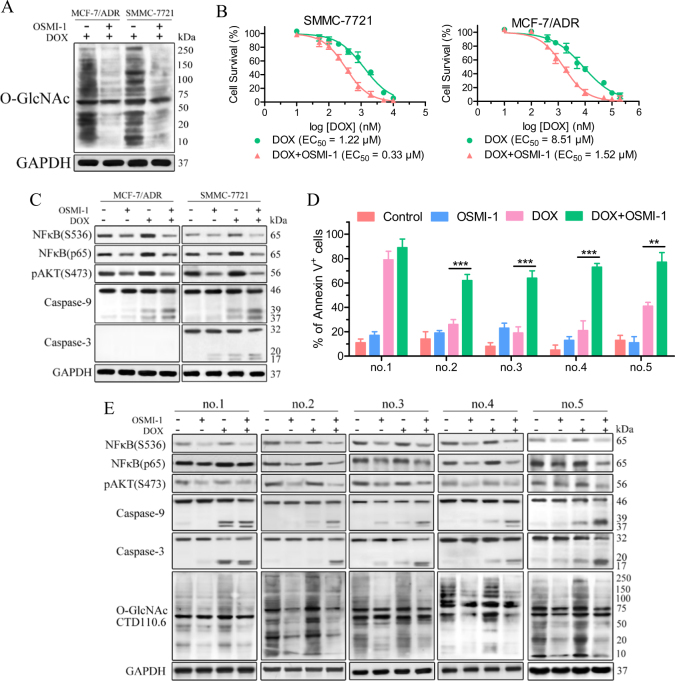
Inhibition of O-GlcNAc sensitizes resistant cancer cells to chemotherapy. **a** SMMC-7721 and MCF-7/ADR cells were treated with 1 μM DOX alone or together with 20 μM OSMI-1 for 6 h. The protein levels were examined by immunoblotting. DMSO was used as a control. **b** SMMC-7721 and MCF-7/ADR cells were treated with increasing doses of DOX alone or together with 20 μM OSMI-1 for 24 h and the cell viability was then assessed though an MTS assay. The EC_50_ values were calculated. **c** SMMC-7721 and MCF-7/ADR cells were treated with 1 μM DOX alone or together with 20 μM OSMI-1 for 6 h. The protein levels were examined by immunoblotting. DMSO was used as a control. **d** and **e** Primary AML cells were treated with 5 μM DOX alone or together with 20 μM OSMI-1 for 24 h. The cells were then stained with Annexin V for flow cytometry analysis. The protein levels were examined by immunoblotting and DMSO was used as a control. **p < 0.01, ***p < 0.001

Blockage of the HBP or downstream O-GlcNAcylation disrupted pro-survival pathways, suggesting that O-GlcNAc suppression is promising in overcoming the resistance of cancer cells to chemotherapy. A western blot analysis showed that DOX-induced cellular O-GlcNAcylation was significantly decreased in SMMC-7721 and MCF-7/ADR cells by treatment with the OGT inhibitor OSMI-1^24^ (Fig. 5a). DOX alone induced SMMC-7721 and MCF-7/ADR cell death with half-maximal effective concentrations (EC_50_) of 1.22 μM and 8.51 μM, respectively (Fig. 5b), whereas no substantial cell killing was observed with OSMI-1 alone (EC_50_ > 100 μM, Fig. S5). With the combination of DOX and OSMI-1 (20 μM), the EC_50_ values for MCF-7/ADR cell death decreased to 1.52 μM (Fig. 5b). This combination of OSMI-1 with DOX also induced a four-fold increase in the efficacy of SMMC-7721 cell killing (DOX EC_50_ = 0.33 μM), which is comparable to the EC_50_ (0.34 μM) for the DOX-sensitive MCF-7 cell line (Fig. S6). Similar potentiation of OSMI-1 combined with CPT or 5-FU was also observed in SMMC-7721 and MCF-7/ADR cells (Fig. S7). Furthermore, 24 h treatment of these cells with the combination of DOX and OSMI-1 resulted in a significant decrease of activated NF-κB and AKT compared with DOX alone. An increase in the level of cleaved caspase-9/3 was also observed in the cells after combinatorial treatment (Fig. 5c). These observations suggest that the inhibition of O-GlcNAc sensitizes resistant cancer cell lines to chemotherapy.

Furthermore, five primary acute myeloid leukemia (AML) patient samples (Table S1) were examined for OSMI-1-induced sensitization to DOX (Fig. 5d). DOX treatment (5 μM) led to significant cell death in a newly diagnosed untreated patient sample (no. 1). In contrast, 5 μM DOX alone mildly reduced the viability of refractory (*n* = 3) and relapsed (*n* = 1) primary cells (no. 2–5), whereas OSMI-1 hardly affected the viability of these samples. Notably, significant enhancement of OSMI-1 activity by treatment combinations with DOX was observed in these samples (no. 2–5). Cellular O-GlcNAcylation upregulation was induced by DOX alone in these samples (no. 1–5), and this upregulation of O-GlcNAcylation was reduced by the combination of DOX with OSMI-1. A decrease in the levels of phosphorylated NF-κB and AKT accompanied with an increase in the levels of activated caspase-9/3 was observed after the DOX/OSMI-1 combination treatment (Fig. 5e). All these results agree with those observed in established cell lines and support a cooperative mechanism between chemotherapy and O-GlcNAc inhibition.

## Discussion

Chemoresistance is the most significant reason for the failure of cancer chemotherapeutics and is crucial to cancer metastasis and recovery^1^, but the interplay between cancer metabolic reprogramming and resistance following chemotherapy is not fully understood. Here we report that the HBP has an important role in the cellular metabolic response to chemotherapy by regulating O-GlcNAcylation, and that this effect has important implications in drug resistance. First, we discovered that drug stress activates the HBP via the AKT/XBP1 axis. Second, O-GlcNAcylation is dynamically induced by the HBP and is required for cell survival and chemoresistance in cancer cells upon drug treatment. This post-translational modification contributes to the inhibition of apoptosis by blocking caspase cleavage and the activation of pro-survival transcription factors, such as NF-κB and AKT, in response to chemotherapy. Furthermore, suppression of the HBP or O-GlcNAcylation reduces cancer cell resistance to chemotherapy. Our studies therefore reveal that O-GlcNAcylation is an important regulator of cell-death thresholds to chemotherapy that coordinates the HBP with chemoresistance.

Among the many hallmarks of cancer, altered metabolism has returned to the forefront as a potential therapeutic target. A neglected but integral branch of glucose metabolism is the HBP, which produces the sugar substrate of O-GlcNAcylation^25^. In this study, cells with intrinsic and acquired resistance were characterized by higher levels of O-GlcNAcylation than sensitive and parental cells, whereas no correlation between cell sensitivities and OGT or OGA expression was found. In addition, O-GlcNAcylated proteins were dynamically increased upon acute treatment with DOX, suggesting that abnormalities in O-GlcNAcylation participates in chemoresistance. The enzyme responsible for this conjugation, OGT, is largely dependent on the cellular free UDP-GlcNAc levels for enzymatic activity. OGT expression levels did not change during drug stress, indicating that the synthesis of UDP-GlcNAc through the HBP is pivotal in regulating O-GlcNAcylation. Significant increases in the GFAT protein levels and activity were detected during DOX treatment, indicating that the increased metabolite levels can potentially enter the HBP. The results showed significant increases in UDP-GlcNAc and cellular O-GlcNAcylation, supporting our hypothesis that the HBP is activated during drug stress. Although UDP-GlcNAc serves as a donor substrate in multiple glycosylation reactions, the regulation of cellular O-GlcNAcylation by OGT knockdown and overexpression significantly sensitized the resistant cells to DOX, suggesting that O-GlcNAcylation has a key role in chemoresistance.

Multiple lines of evidence suggest that O-GlcNAcylation is increased in various cellular stresses, and that this increase might be protective^26,27^. Our work extends the function of O-GlcNAcylation in modulating cell resistance to chemotherapy. Apoptosis-related caspase-9/3 were stimulated by drug-induced O-GlcNAcylation. This modification blocks the cleavage/activation of caspase-9/3 and serves as a potential anti-apoptotic mechanism. In addition, the O-GlcNAcylation of pro-survival transcription factors (NF-κB and AKT) promotes their phosphorylation, which, in turn, enhances their pro-survival function. AKT activation further forms a positive feedback loop that strengthens the flux through the HBP and regulates genotoxic resistance in cancer cells. These O-GlcNAcylation-triggered pro-survival factors strengthen the thresholds that need to be overcome prior to the initiation of cell death pathways. However, our results do not exclude the possibility that other pro-survival processes, such as autophagy and mitochondrial dysfunction, could participate, together with HBP-induced O-GlcNAcylation, in determining the fate of cancer cells in response to chemotherapy treatment. Given the multiple targets of O-GlcNAcylation in epigenetic regulation, obtaining a more in-depth understanding of the precise roles of this modification in chemoresistance and cell survival requires further investigation.

Increased activity of the PI3K/Akt pathway was previously shown to participate in resistance to various chemotherapeutic agents^28^. We observed that DOX treatment significantly increased AKT phosphorylation in SMMC-7721 and MCF-7/ADR cells. Pharmacological inhibition of AKT using MK2206 significant reduced the XBP1 expression level and restored the sensitivity of SMMC-7721 and MCF-7/ADR cells to DOX. A previous study indicated that the transcription factor XBP1 can directly promote the transcription of GFAT^20^. Indeed, XBP1 deficiency decreased GFAT expression in both SMMC-7721 and MCF-7/ADR cells in response to DOX treatment. These data uncover a mechanistic axis that directly links the HBP, O-GlcNAcylation, and chemoresistance under drug stress.

The therapeutic efficacy of first-line anticancer drugs, such as DOX, CPT and 5-FU, is limited by the resistance developed during the treatment. We demonstrated that the OGT inhibitor OSMI-1 effectively sensitized DOX-resistant SMMC-7721 and MCF-7/ADR cells to DOX and 5-FU. For the uracil analogue 5-FU, although it is an analogue of uracil, 5-FU may not affect the UDP-GlcNAc synthesis in this study. The inhibition of O-GlcNAcylation was also proven to serve as a useful strategy for DOX resistance in primary refractory or relapsed AML cells, suggesting that the combination of an O-GlcNAcylation inhibitor with chemotherapy could be a novel strategy in cancer therapy. However, although pharmacological targeting of OGT is possible in vitro, important work is still required for in vivo application.

**Fig. 6:**
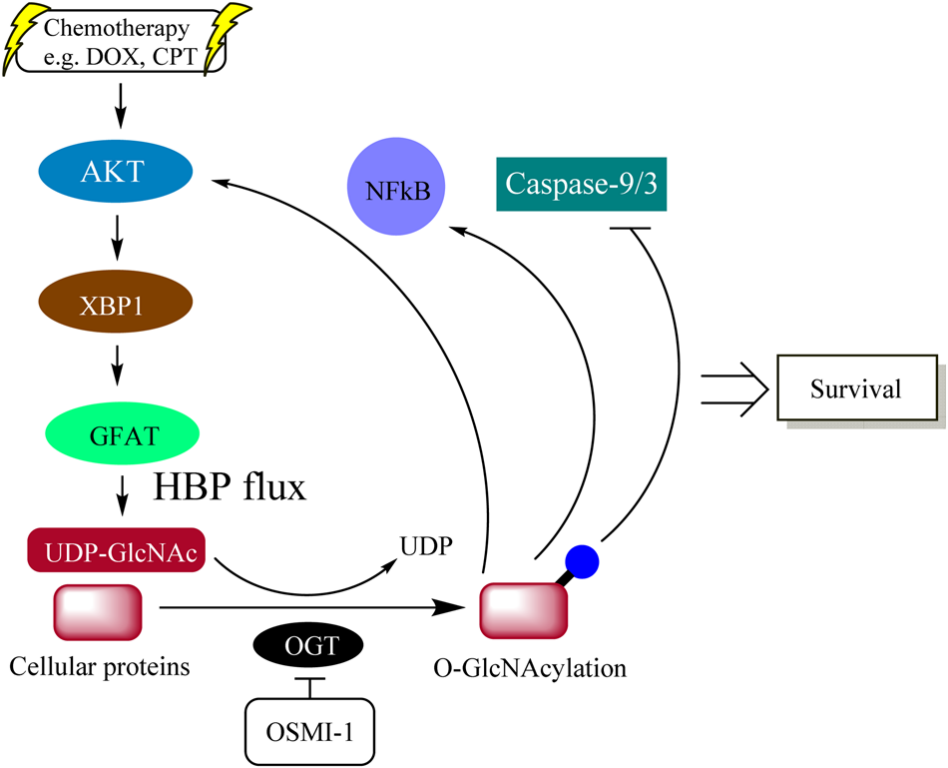
Schematic working model for cell survival signalling based on chemotherapeutic drug-induced activation of the HBP and O-GlcNAcylation. Chemotherapeutic drugs, such as DOX and CPT, stimulate AKT/XBP1 and then induce GFAT through upregulation of the HBP and O-GlcNAcylation. Excess O-GlcNAc leads to cell survival by inhibiting the apoptotic function of caspases and activating pro-survival transcription factors, such as NF-κB and AKT

Taken together, our results provide evidence showing that activation of O-GlcNAcylation by increasing flux through the HBP impacts cell survival and resistance during chemotherapy (Fig. 6). We propose that this is an important physiological response of cancer cells stimulated with chemotherapy. By promoting cell survival, HBP-induced O-GlcNAcylation might have an important role in determining cell fate and maintaining cellular immortality following drug treatment.

## Materials and methods

### Cell culture and reagents

MCF-7, HL60, Hela and SMMC-7721 cells were obtained from Type Culture Collection of the Chinese Academy of Sciences (Shanghai, China) and were used within 6 months from resuscitation. All the cells were cultured in 90% RPMI-1640 (Gibco, USA) supplemented with 1 % penicillin/streptomycin antibiotics (Gibco) and 10 % fetal bovine serum (Gibco). DOX (Sigma, St Louis, MO, USA) was added to cell cultures in stepwise increasing concentrations from 0.1 to 10 μM for 4 months to develop an ADR subline, namely HL60/ADR and MCF-7/ADR, correspondingly. To maintain the ADR phenotype, the complete medium of the resistant cell clones were supplemented with 2 μM DOX. ADR cells were maintained in complete medium without adriamycin for 1 week and cells with > 90% viability before subsequent treatments.

Human OGT complementary DNA was cloned in pcDNA3.1 plasmid. AZA, VST, MK2206 and CPT were purchased from Sigma. Lipofectamine 2000, Annexin V-FITC/PI and G418 were from Invitrogen (Grand Island, NY, USA).

### Isolation and culture of AML primary cells

Cells from five patients (refractory (*n* = 3), relapsed (*n* = 1) and newly diagnosed untreated (*n* = 1)) diagnosed with AML (except M3) according to the World Health Organization classification were studied. Refractory patients failed to achieve remission following at least two induction chemotherapy. Relapsed patient recurred after complete remission ( < 5% marrow blasts) and resisted to the re-induction protocol. Tumour cells were obtained from bone marrow and the characteristics of these patients are listed in Supplementary Table S1. In all cases, informed consent was obtained in accordance with the guidelines and the approval of the Second Affiliated Hospital of Dalian Medical University (Dalian, Liaoning, China) and the Declaration of Helsinki.

Mononuclear cells from peripheral blood samples were isolated by Ficoll-Hypaque sedimentation (Sigma Chemical Co., St Louis, MO, USA). Contaminating red cells were lysed in 0.8% ammonium chloride solution for 10 min. The cells were either used directly or cryopreserved in liquid nitrogen in the presence of 10% dimethyl sulfoxide and 60% fetal calf serum (Gibco BRL, Grand Island, NY, USA). Cells from AML patients were cultured in RPMI 1640 culture medium supplemented with 10% fetal bovine serum and 1% penicillin/streptomycin antibiotics.

### Quantitative RT-PCR

Total RNA was isolated using the Trizol method (Invitrogen). A total of 5 μg of RNA were reverse-transcribed and amplified using One Step SYBR PrimeScript PLUS RT-PCR Kit (TaKaRa, Dalian, China) and the Thermal Cycler Dice instrument (TaKaRa) according to the manufacturer’s instructions. RT-PCR primers for GADPH and GFAT are listed as follows: GADPH sense: 5′- TGGTGAAGCAGGCATCTGAG-3′, antisense: 5′- CTCCTGCGACTTCAACAGCA-3′; GFAT sense: 5′- AACTACCATGTTCCTCGAACGA-3′, antisense: 5′- CTCCATCAAATCCCACACCAG-3′. Results were normalized to GAPDH.

### Immunoblotting and immunoprecipitation

The cell lysing, western blotting and immunoprecipitation were performed as previously described^29^. The following antibodies were used: OGT, OGA, GAPDH, P-gp, AKT (Rabbit), XBP1, GFAT, PFK and G6PD were from Abcam (Hong Kong, China). pAKT (S473), AKT (mouse), GRP78, Caspase-9 and Caspase-3 were from Cell Signaling Technology (MA, USA); O-GlcNAcylation antibody (CTD110.6) was from BioLegend (MA, USA). Chemiluminescent detection was performed using ECL kit (GE Healthcare, USA).

### Cell viability and apoptosis assays

Cells were treated for different times in 96-well culture plates in 200 μL of culture medium. Each concentration was tested in duplicate at least thrice separately. The viable cells were determined using the CellTiter 96 AQueous non-radioactive cell proliferation MTS assay (Promega) and the inhibition rates were analysed using GraphPad 5. Cell apoptosis was assessed by Annexin-V/PI staining using a FACSCalibur flow cytometry system.

### Gene silencing and stable transfection

Gene silencing was achieved though transfection with siRNA using the Lipofectamine 2000 transfection reagent following the manufacturer’s instructions. OGT siRNA (sc-40780), GFAT siRNA (sc-6068), XBP1 siRNA (sc-38627), GRP78 siRNA (sc-29338) and scrambled siRNA (sc-37007) were obtained from Santa Cruz Biotechnology (CA, USA).

Transfection of the MCF-7 cell line was performed with Lipofectamine according to the manufacturer’s instructions. Under our conditions, 30–40% of cells are routinely transfected. The stably transfected cells were then selected by the addition of 800 μg/mL geneticin (G418), which was purchased from Invitrogen, to the medium. After 3 weeks, the selected stably transfected cells were further cultured with G418 at a concentration of 400 μg/mL and OGT-overexpressing clones were selected for other experiments.

### Statistical analysis

All data are presented as the mean ± SEM, *N* = 3. Data groups were compared by two-tailed Student’s *t*-test using the GraphPad Software. Differences between groups were considered statistically significant if *p* < 0.05. The statistical significance is denoted by asterisks (*p < 0.05; **p < 0.01; ***p *<* 0.001).

## Electronic supplementary material

Supporting information
