# Supplementary material for: O-GlcNAc elevation through activation of the hexosamine biosynthetic pathway enhances cancer cell chemoresistance
Source: Cell Death Dis. 2018 Apr 30;9(5):485. doi: 10.1038/s41419-018-0522-0 (PMC5924752; doi:10.1038/s41419-018-0522-0)
Supplement: Supplementary file 1 — Supporting information [file 41419_2018_522_MOESM1_ESM.docx]

Supporting information

**Methods**

*Liquid chromatography-tandem MS (LC-MS/MS) for UDP-GlcNAc quantification.*

UDP-GlcNAc is a strongly polar and ionic compound that is not well retained on most reversed-phase high performance liquid chromatography (HPLC) columns. In addition, UDP-GlcNAc may react with some metal ions and tends to form adducts and multiple-charged substances that cannot facilitate the determination of UDP-GlcNAc by liquid chromatography-tandem mass spectrometry (LC-MS/MS) method. As a result, the analysis of UDP-GlcNAc in biological samples has always been challenging in terms of both sample purification and detection. With rare exceptions, derivatization is necessary for the determination of UDP-GlcNAc in biological samples. In this study, UDP-GlcNAc and its chiral isomer UDP-GalNAc were derivatizated with trimethylsilyldiazomethane before chromatographic separation.

The derivatization reagent was prepared in dark conditions just before the derivatization procedure. A 3.0-mL aliquot of trimethylsilyldiazomethane was added to 9.75 mL of methanol:water (3:0.25; v/v) under nitrogen, mixed immediately, and stored under nitrogen in dark conditions.

Cells were subjected to ultrasonication (4 × 30 s on half-power) in methanol: water (12:1; v/v), The homogenate was then centrifuged at 10000 g, 4 °C for 10 min. All the samples were spiked with 10 μM probenecid as internal standard. The supernatants containing the UDP-GlcNAc were collected. Then 100 μL supernatant, 10 μL internal standard probenecid (100 ng/mL) and 100 μL derivatization reagent were added to glass tubes, and reacted for 30 min and dried at 40 °C under a stream of nitrogen in a TurboVap evaporator (Zymark Corp., MA, USA). The residues were reconstituted in 500 μL of the mobile phase, and 10 μL of the sample was injected into the LC-MS/MS system for analysis.

The LC-MS/MS system comprised an UPLC system (Waters Corporation, MA, USA) and Qtrap 6500 mass spectrometer (SCIEX, Toronto, Canada) equipped with an electrospary ionization source in the negative ion mode. The analysis of UDP-GlcNAc was performed on a Cosmosil NH_2_ MS column (250 mm×4.6 mm i.d., 5 μm; Cosmosil 5NH_2_-MS, Nacalai Tesque Inc. , Japan) protected by a Security Guard C18 guard column (4 mm×3.0 mm i.d., Phenomenex, Torrance, CA, USA). The mobile phase was composed of 40% A (1 mM ammonium acetate) and 60% B (acetonitrile with 1 mM ammonium acetate). The ion spray voltage was adjusted to 4500 V, and the source temperature was set at 500 °C. The curtain gas, gas 1, and gas 2 were nitrogen set at 35, 50, and 50 psi. Multiple reaction monitoring (MRM) was used for detection. MRM scan mode was used to monitor transitions at m/z 648.0 → 296.0 for UDP-GlcNAc derivative and UDP-GalNAc derivative, and m/z 283.9 → 239.9 for internal standard probenecid derivative. The declustering potentials (V) for UDP-GlcNAc, UDP-GalNAc and probenecid were -60, -60 and -50, respectively; and collision energies (eV) were -40, -40 and -20, respectively. Data acquisition and integration were controlled by Applied Biosystems Analyst version 1.6 software. As a result, the retention time for UDP-GlcNAc derivative, UDP-GalNAc derivative and probenecid were 19.4 min, 20.5 min and 12.5 min, respectively. For UDP-GlcNAc quantification, a calibration curve was constructed by analyzing a series of UDP-GlcNAc derivative dilutions spiked with 10 μM Probenecid according to the aforementioned chromatographic method.

*GFAT enzyme activity*

GFAT activity was measured as described previously [^1^](#_ENREF_1). Cells were lysed using extraction buffer (60 mM KH_2_PO_4_, pH 7.0, 1 mM EDTA, and 1 mM dithiothreitol) at 4 °C. The assay mixture contained 15 mM D-fructose-6-phosphate and 15 mM L-glutamine in extraction buffer with 2 mg/mL protein extract. The mixture was incubated at 37 °C for 1 h, and the reaction was terminated by heating at 100 °C for 2 min. After cooling and centrifugation, the end-product D-glucosamine-6-phosphate was estimated through derivatization by ortho-phthalaldehyde and as a fluorimetry read-out. GFAT activity is represented as pmol/mg protein/min.

RT-PCR

cDNA was synthesized from 200 ng RNA using qScript cDNA Supermix (Quanta Biosciences). The cDNA was diluted 1:20, and 5 μL was used per reaction for PCR in a 50 μL reaction volume using Platinum Taq polymerase (Life Technologies) according to the manufacturer’s protocol. The primers used were *XBP1* forward primer, CCTTGTAGTTGAGAACCAGG, and *XBP1* reverse primer, GGGGCTTGGTATATATGTGG. The primers used were *GAPDH* forward primer, GAAGGTGAAGGTCGGAGTC, and *GAPDH* reverse primer, GAAGATGGTGATGGGATTTC. The cycling conditions were 94°C for 2 min, followed by 32 cycles of 94°C for 30 s, 60°C for 30 s, and 72°C for 30 s; 6_ loading buffer was added to the product, and electrophoresis was performed on 10 μL per sample in a 2% agarose gel containing GelRed nucleic acid stain (Biotium).

**References**

1. Zheng J, Khalil M, Cannon JF. Glc7p Protein Phosphatase Inhibits Expression of Glutamine-Fructose-6-phosphate Transaminase from GFA1. *J Biol Chem* 2000, **275**(24)**:** 18070-18078.

Figures


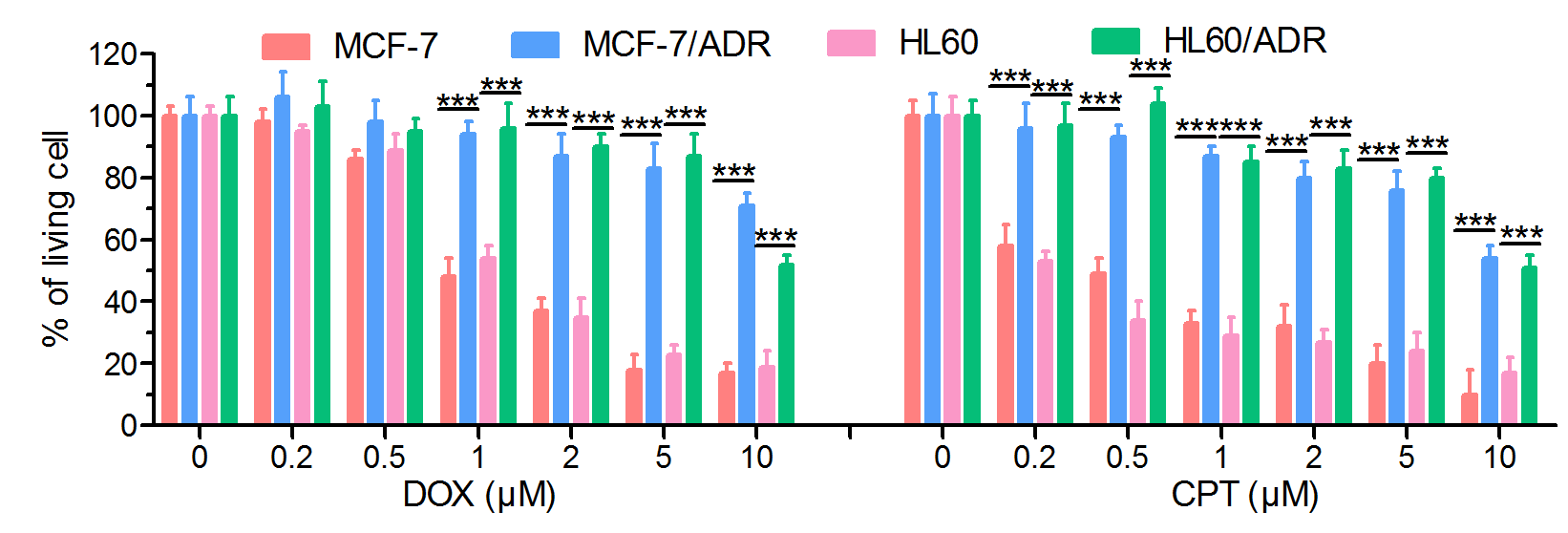
Fig. S1

A

B

Fig. S1 (A) MCF-7, MCF-7/ADR, HL60 and HL60/ADR cells were treated with indicated dose of DOX or CPT for 24 h. Cell viability was assessed by MTS assay. (B) MCF-7, MCF-7/ADR, SMMC-7721 cells were treated with indicated dose of 5-FU for 24 h. Cell viability was assessed by MTS assay. The data represent the means ± SEM, N = 3, ** *p* < 0.01, ****p* < 0.001.

Fig. S2

A


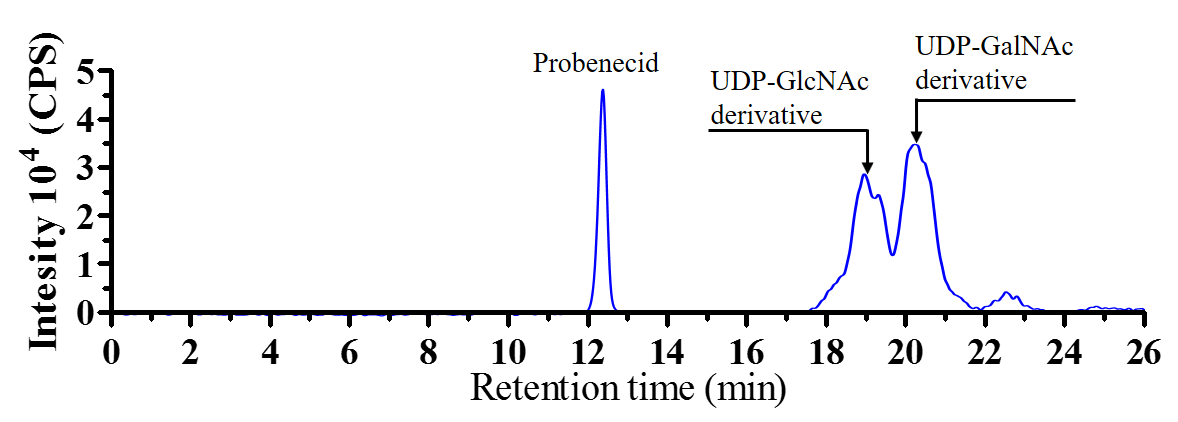


B


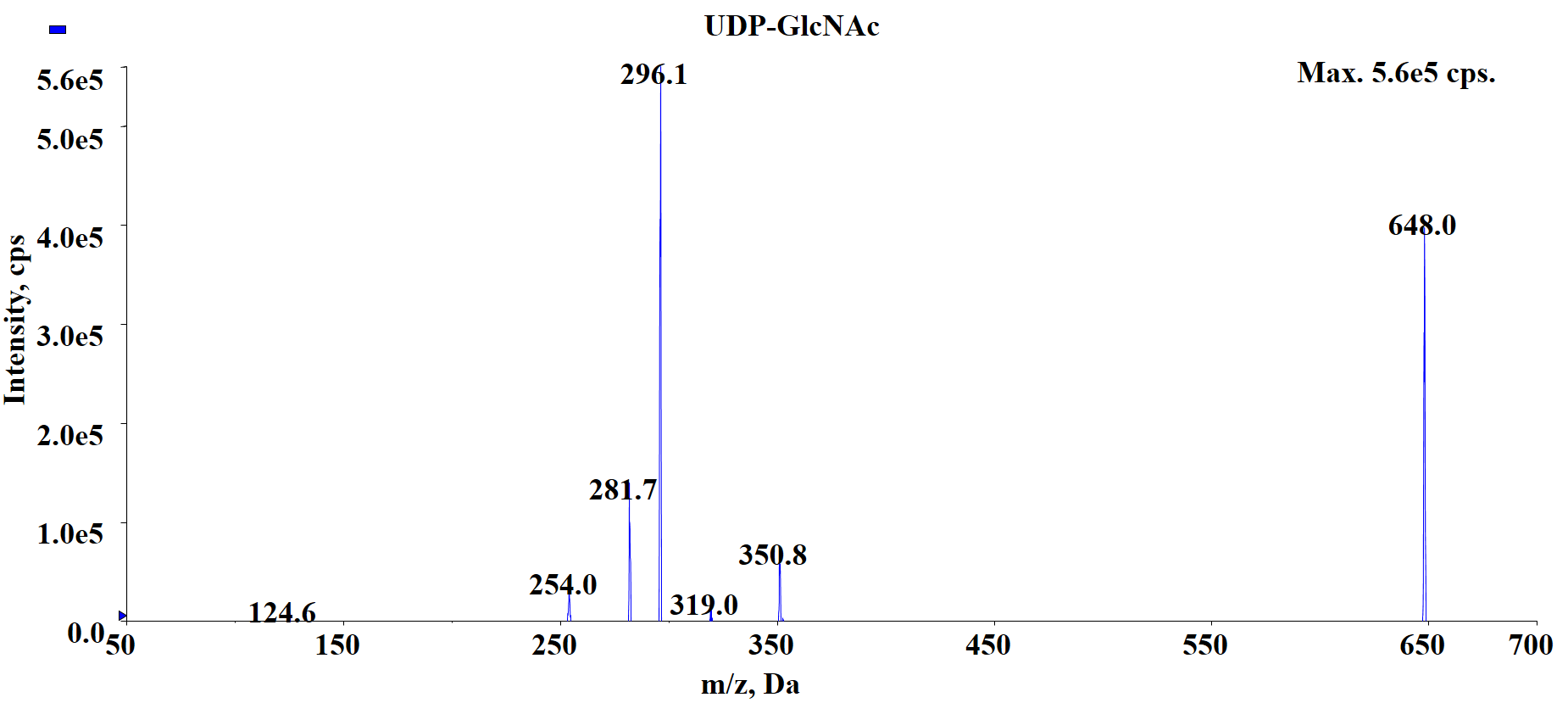


Fig. S3 (A) Separation of UDP-GlcNAc (10 μM), UDP-GalNAc (10 μM) and probenecid (10 μM) by using liquid chromatography. (B) Mass spectrum of UDP-GlcNAc derivative peak from chromatogram of control cell extract.

Fig. S3


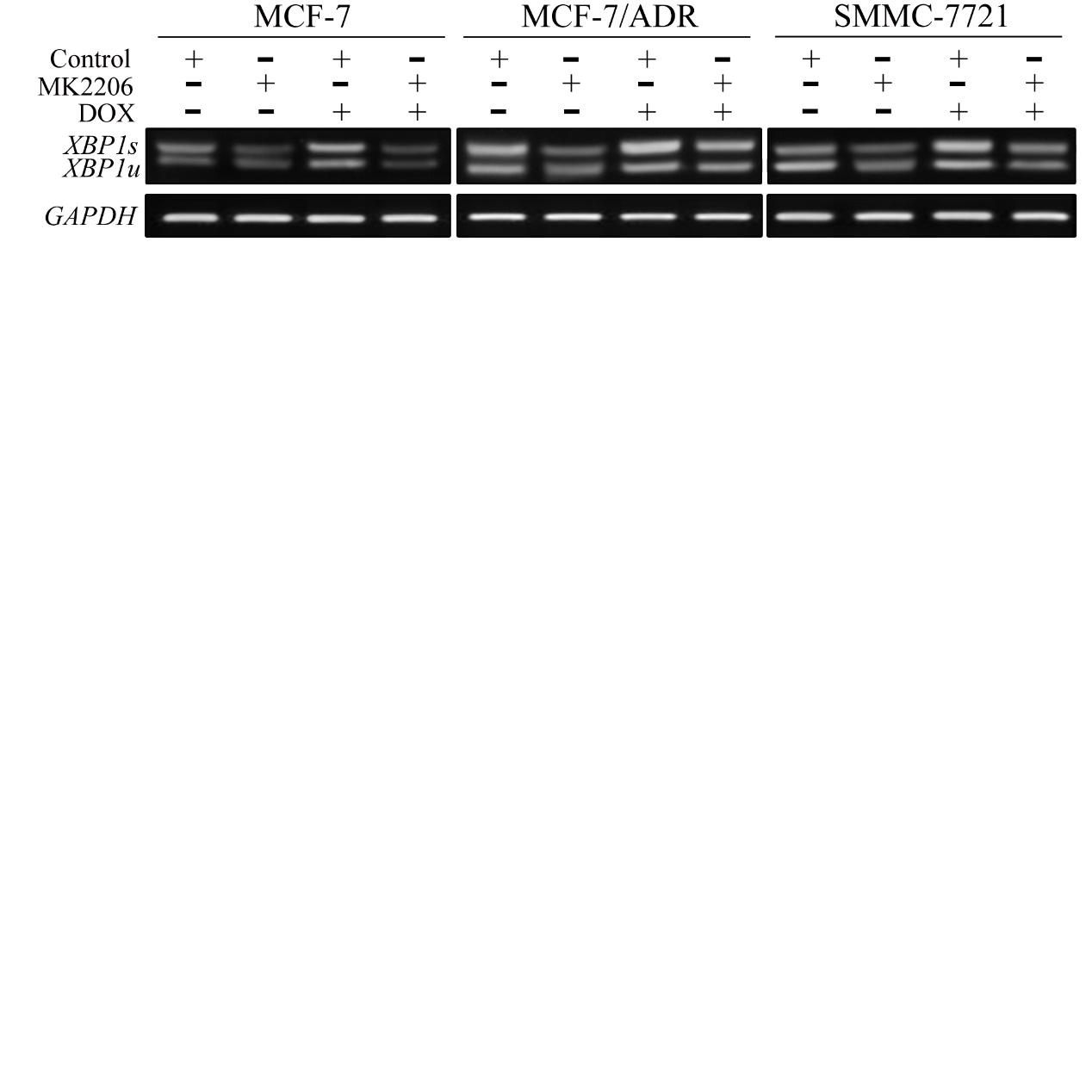


Fig. S3 Representative agarose gel electrophoresis of the RT-PCR product surrounding the *XBP1* splice site in the samples used for Fig. 3A. The indicated cells were treated with DOX (0.1 μM for MCF-7, 1 μM for MCF-7/ADR and SMMC-7721) alone or together with 1 μM MK2206 for 6 h. DMSO was used as a control. *GAPDH* was used as control.

Fig. S4

A


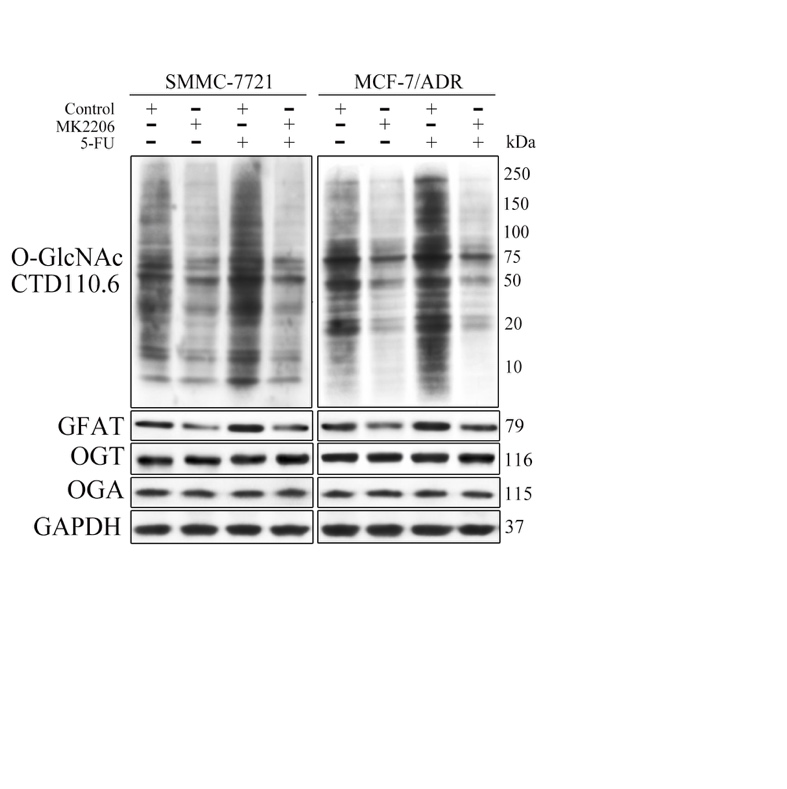

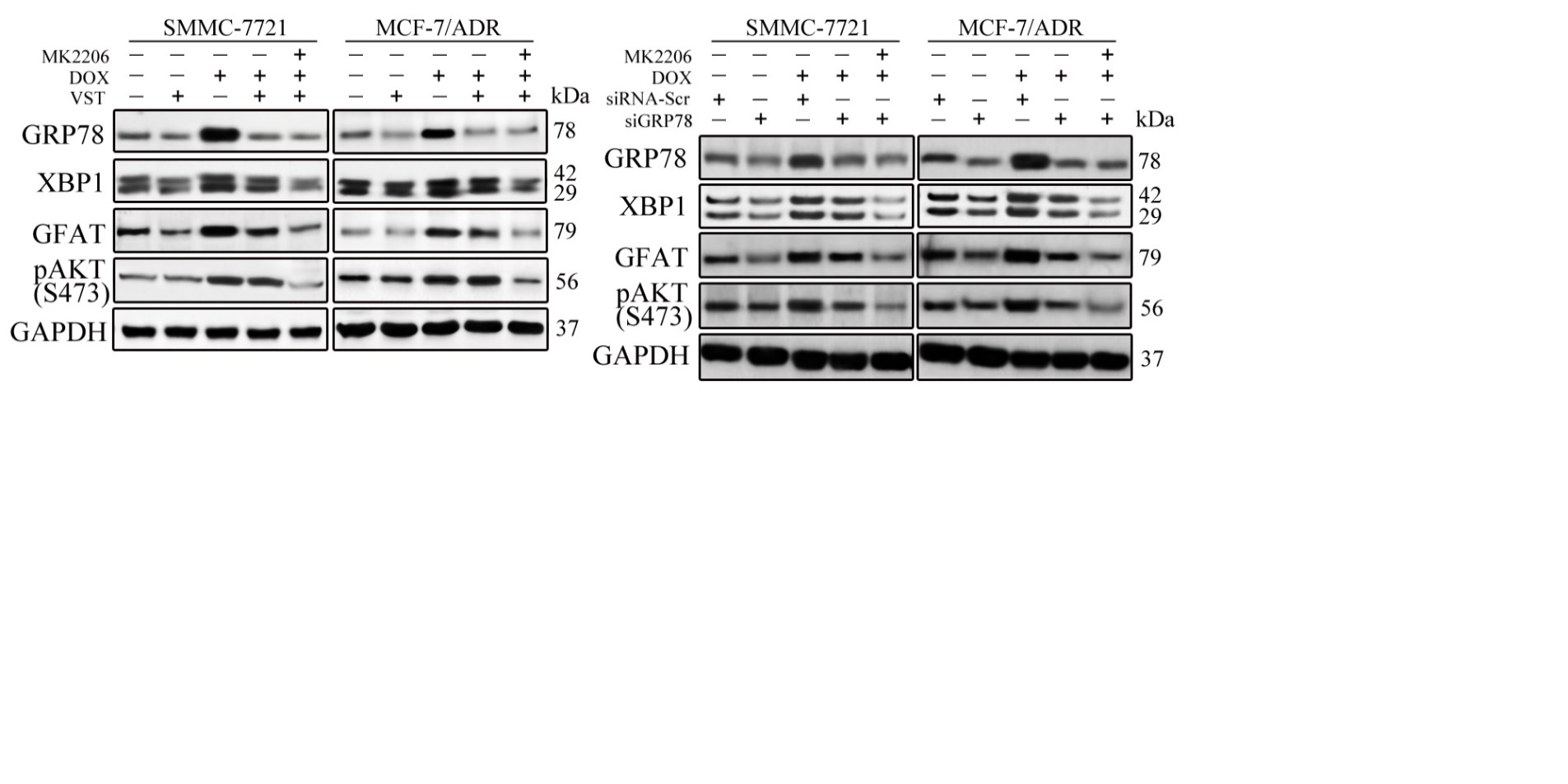


B

Fig. S4 (A) The indicated cells were treated with 5-FU (1 μM for MCF-7/ADR and SMMC-7721) alone or together with 1 μM MK2206 for 6 h. DMSO was used as a control. The protein levels were examined by immunoblotting. (B) AKT/XBP1 axis regulates GFAT expression in a UPR-independent manner. Indicated cells were transfected with scrambled siRNA (siRNA-Scr) or GRP78 siRNA (siGRP78) for 48 h and then treated with 1 μM DOX alone or together with 1 μM MK2206 for 6 h. DMSO was used as a control. Protein levels were examined by immunoblot.

Fig. S5

Fig. S5 SMMC-7721 and MCF-7/ADR cells were treated with increasing doses of OSMI-1 for 24 h, and then cell viability was assessed by MTS assay.

Fig. S6

Fig. S6 MCF-7 and HL60 cells were treated with increasing doses of DOX for 24 h, and then cell viability was assessed by MTS assay.

Fig. S7

Fig. S7 SMMC-7721 and MCF-7/ADR cells were treated with increasing doses of indicated drugalone or together with 20 μM OSMI-1 for 24 h, and then cell viability was assessed by MTS assay. EC_50_ values were calculated.

Table S1

Table 1: Characteristics of patients with AML (n=5)

| Patient | Age/Sex | FAB | Karyotype | Disease status | BM Blast (%) | P-gp |
| --- | --- | --- | --- | --- | --- | --- |
| no. 1 | 58/M | M4 | 46XY | Refractory | 78 | + |
| no. 2 | 42/F | M4 | 46XX | Refractory | 82 | + |
| no. 3 | 22/F | M5 | 46XX | Relapse | 74 | + |
| no. 4 | 51/M | M2 | t(8, 21) | Refractory | 91 | + |
| no. 5 | 32/F | M5 | 46XX | Newly diagnosed | 87 | - |

Abbreviations: AML, acute myeloid leukemia; P-gp, P-glycoprotein; BM, bone marrow; FAB, French-American-British
